# GPRC5A: An Emerging Biomarker in Human Cancer

**DOI:** 10.1155/2018/1823726

**Published:** 2018-10-17

**Authors:** Xiaoxia Jiang, Xin Xu, Mengjie Wu, Zhonghai Guan, Xingyun Su, Shitu Chen, Haiyong Wang, Lisong Teng

**Affiliations:** ^1^Cancer Center, The First Affiliated Hospital, College of Medicine, Zhejiang University, Hangzhou, Zhejiang, China; ^2^Key Laboratory of Precision Diagnosis and Treatment for Hepatobiliary and Pancreatic Tumor of Zhejiang Province, Hangzhou, Zhejiang, China; ^3^Department of Pediatric Surgical Oncology, Children's Hospital, Zhejiang University, Hangzhou, Zhejiang, China

## Abstract

Aberrant expression of G protein-coupled receptors (GPCRs) is frequently associated with tumorigenesis. G Protein-coupled receptor class C group 5 member A (GPRC5A) is a member of the GPCR superfamily, is expressed preferentially in lung tissues, and is regulated by various entities at multiple levels. GPRC5A exerts a tumor suppressive role in lung cancer and GPRC5A deletion promotes lung tumor initiation and progression. Recent advances have highlighted that GPRC5A dysregulation is found in various human cancers and is related to many tumor-associated signaling pathways, including the cyclic adenosine monophosphate (cAMP), nuclear factor (NF)-*κ*B, signal transducer and activator of transcription (STAT) 3, and focal adhesion kinase (FAK)/Src signaling. This review aimed to summarize our updated view on the biology and regulation of GPRC5A, its expression in human cancers, and the linked signaling pathways. A better comprehension of the underlying cellular and molecular mechanisms of GPRC5A will provide novel insights into its potential diagnostic and therapeutic value.

## 1. Introduction

The early and accurate diagnosis of cancer is a long-standing problem which, if solved, can significantly improve the patient prognoses. For this purpose, modern molecular diagnosis is an advanced and essential detection technique. As cancer is the result of the accumulation of adverse disease-related molecular events, it is reasonable to stratify patients according to genetic alterations in one or more genes. This has become an important factor in clinical intervention [1, 2]. However, while a number of cancer biomarkers for molecular diagnosis have been described recently, the specificity and diagnostic capacity of currently available biomarkers are limited [3–5]. Therefore, there remains a requirement for novel biomarkers with high specificity and sensitivity.

G protein-coupled receptors (GPCRs) are one of the largest and most diverse superfamilies of receptors and play a key role in a broad variety of physiological processes [6]. GPCRs are characterized by a common structure of one bundle of seven transmembrane helices connected by three extracellular and three intracellular loops. The vast majority of ligands interact with the extracellular oriented part of the helices [7, 8]. Due to their broad physiological functions, aberrant GPCRs activation is frequently associated with disease initiation and progression. A number of studies have indicated the critical role of GPCRs in tumor proliferation, invasiveness, angiogenesis, metastasis, and drug resistance [9–16]. Notably, GPCRs are highly attractive targets in drug design, accounting for more than 30% of all commercially available pharmaceutical drugs [17, 18]. Recently, G protein-coupled receptor family C group 5 member A (GPRC5A), a member of class C orphan GPCRs, has been found to be dysregulated in several human cancers. It has been shown to have an important effect on tumor progression [19]. This review will focus on recent advances in GPRC5A research and on its role in human cancer.

## 2. The GPCR Family C Group 5

**Figure 1:**
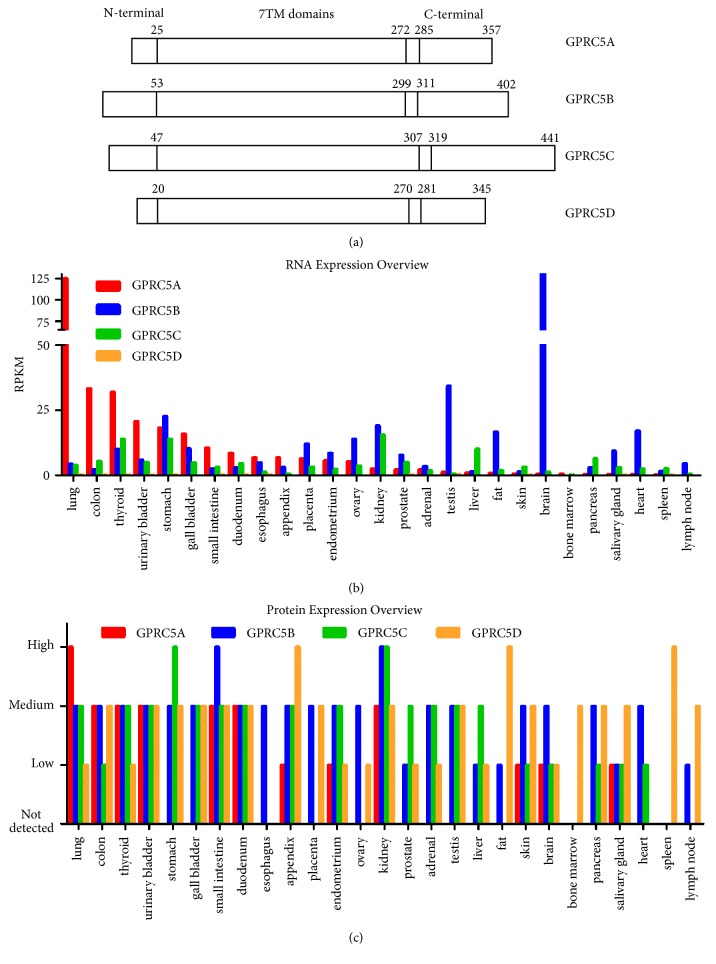
**(a) **The protein structure of GPRC5A–D. The amino acids of the four proteins are detailedly numbered. They have similar length of 7TM domain and have short N-ternimal of 20-53 amino acids as detailed in the text.** (b) **The mRNA expression profile of GPRC5A–D in different organs. Data was compiled from the RNA sequence conducted by Fagerberg L. et al.** (c) **The protein expression levels of GPRC5A–D (Data from the Human Protein Atlas http://www.proteinatlas.org/).

GPRC5A, also known as retinoic acid-induced protein 3 (RAI3) or retinoic acid-inducible gene (RAIG) 1, is a member of class C orphan GPCRs. GPRC5A was first described in the UMSCC-22B cell line as an all-transretinoic acid- (ATRA-) responsive gene [16], is located on 12p13.1, and encodes a 40 kDa protein. Three other members of this group, namely, GPRC5B (also known as RAIG2), GPRC5C (also known as RAIG3), and GPRC5D, were consequently identified [16, 20–22]. The four proteins share 31–42% amino acid sequence identity and have high sequence similarity within their transmembrane domains [22]. GPRC5A, GPRC5B, and GPRC5C can be induced by retinoic acid (RA) in a concentration- and time-dependent manner, whereas GPRC5D cannot. Unlike other members of the C family of GPCRs, whose ligand binding sites are located within the large N–terminal domain, the four members of group 5 possess very short N-terminals of 30–50 amino acids. Instead of binding to the N terminal domain, agonists can bind to the 7TM domains of the four proteins [22, 23]. The protein structure of GPRC5A–D is summarized in Figure 1(a). Interestingly, GPRC5A–D are expressed in a tissue-specific manner with GPRC5A being preferentially expressed in lung tissues; GPRC5B is predominately localized in tissues of the central nervous system, while GPRC5C and GPRC5D are observed in a variety of tissues (Figures 1(b) and 1(c)) [21, 22, 24].

## 3. Regulation of GPRC5A Expression

### 3.1. Transcriptional Regulation

As summarized in a previously published review, the* GPRC5A* gene has many transcription factor binding sites; among these, RA is the most well studied. RA is a vitamin A-derived morphogen with many effects on cell growth and differentiation [25, 26].* GPRC5A *has a RA response element (RARE) in its 5' upstream region, which binds the RA receptor (RAR)/retinoid X receptor (RXR) heterodimer. In the presence of RA, the inhibitory effect of the RAR/RXR heterodimer on the transcription of* GPRC5A* is relieved, resulting on the transcription of the gene [27, 28].

A custom-made cDNA microarray analysis showed that GPRC5A expression is induced when the levels of cyclic adenosine monophosphate (cAMP) increase. Specifically, a cAMP-responsive element (CRE) motif exists close to the transcription initiation site of* GPRC5A.* By upregulating cAMP levels, forskolin induces* GPRC5A *transcription and this effect can be strengthened by RA [29].

Furthermore, microarray and quantitative polymerase chain reaction (qPCR) assays conducted in four p53-mutant cell lines (MDA-MB-468, BT-20, BT-549, and SK-BR-3) and four p53 wild-type cell lines (MCF-7, T47D, ZR-75-1, and BT-474) have demonstrated that GPRC5A is a target of P53 and is suppressed by wild-type p53 [30]. In the same study, chromatin immunoprecipitation (ChIP) assays indicated that p53 binds to GPRC5A in a sequence-specific way in human ovarian tumor cell line 2774qw1. Additionally, overexpression of p53 in the* p53*-null human nonsmall cell lung cancer (NSCLC) H1299 cells led to increased GPRC5A expression, while p53 knockdown in the* p53* wild-type human NSCLC A549 cells resulted in decreased expression of GPRC5A, indicating that GPRC5A is involved in the antitumor effect of p53 in NSCLC cells [31].

Additionally, GPRC5A expression is also related to BRCA1 status. In breast tumors with wild-type BRCA1, GPRC5A expression is higher than in BRCA1-mutated tumors.* In vitro* experiments show that knockdown of BRCA1 results in decreased expression of GPRC5A in MDA-MB-231 breast cancer cells, while the opposite results are obtained with BRCA1 overexpression [32].

### 3.2. Post-Transcriptional Regulation

Little is currently known about the post-transcriptional regulation of* GPRC5A*. MicroRNAs (miRNAs), small noncoding RNA molecules that regulate the expression of target genes in a sequence-dependent way, are important post-transcriptional regulators [33, 34]. A computational analysis conducted in a previous review using the RNA22 algorithm indicated that there are many putative miRNAs targeting* GPRC5A*, specifically 343 in the 5'untranslated region (UTR), 595 in the coding sequence (CDS), and 1170 in the 3' UTR [19]. Of these, miR-103a-3p has been extensively studied. miR-103a-3p has two target sites in the 5'UTR of* GPRC5A*, and* in vitro *studies have found that it suppresses the expression of* GPRC5A* mRNA and protein by binding to either of them [35]. Besides, miR-204 can inhibit GPRC5A expression via binding to its 3' UTR in gastric cancer (GC) [36].

RNA binding proteins (RBPs) also participate in the posttranscriptional regulation of GPRC5A. HuR, an RBP encoded by the* ELAVL1* gene, was identified to upregulate GPRC5A expression via mRNA stabilization by binding to the 3' UTR of GPRC5A [37, 38]. Other crucial posttranscriptional regulators such as long noncoding RNAs (lncRNAs) are thought to significantly impact the regulation of GPRC5A, but evidence remains lacking [39–41].

### 3.3. Post-Translational Modification of GPRC5A

GPRC5A has several phosphorylation sites which have been found to be involved in many biological processes. Phosphorylation of serine (SER) 301 and 345 takes place during mitosis [42, 43]. The phosphorylation of GPRC5A in two conserved double-tyrosine (TYR) motifs, TYR-317/TYR-320, and TYR-347/TYR-350 is mediated by epidermal growth factor receptor (EGFR), leading to inactivation of the protein's tumor suppressive function [44, 45]. Furthermore, sequence analysis predicts that the arginine (ARG) 158 site of GPRC5A can be N-glycosylated. Additionally, several studies indicate that GPRC5A can be ubiquitinated at a number of sites, although the details remain to be clarified [46–53].

## 4. GPRC5A and Downstream Signaling Pathways

### 4.1. cAMP Signaling Pathway

GPRC5A is one of several genes whose expression increases when the cAMP level is elevated. As mentioned above, cAMP binds to the CRE motif in the* GPRC5A* gene inducing its transcription. In the same study, the authors found that, in the human thyroid epithelial cell line Nthy, GPRC5A expression is negatively correlated with intracellular cAMP and Gs alpha levels and that the suppression of GPRC5A results in inhibition of cell growth and induction of apoptosis [29]. These results suggest that there exists a negative feedback loop between cAMP and GPRC5A that also involves Gs alpha.

### 4.2. Nuclear Factor- (NF-) *κ*B Signaling Pathway

NF-*κ*B controls the expression of genes involved in many biological and pathological processes, and plays a critical role in inflammation and tumorigenesis. Dysregulation of NF-*κ*B is related to pathological alterations in various cells including epithelial and stromal cells [54, 55].* In vivo* studies have demonstrated that* GPRC5A* knockout mice are more sensitive to lipopolysaccharide- (LPS-) induced NF-*κ*B signaling activation than are GPRC5A wild-type mice and that they have lower levels of proinflammatory cytokines and chemokines. Moreover,* in vitro* studies showed that GPRC5A knockout cells produce higher levels of chemokines and cytokines and promote broader macrophage migration through their conditioned medium compared to GPRC5A wild-type cells in a NF-*κ*B dependent manner [56]. Additionally, they found that selective inhibition of NF-*κ*B through the expression of the superrepressor IkBa in the GPRC5A knockout mice significantly alleviates the inflammation response and mice lung injury induced by LPS [57]. However, all of these results were based on the deletion of GPRC5A, and further in-depth studies are warranted to further explore the relationship.

### 4.3. Signal Transducer and Activator of Transcription (STAT) 3 Signaling Pathway

STATs are transcription factors that regulate cell growth, differentiation, survival and development by mediating the expression of target genes [58]. STAT3 is the best studied member of the STAT family. Aberrant activation of STAT3 has been identified in various human cancers, and correlates with poor prognosis in gastric, breast and lung cancer [59–65]. Recent studies suggest that GPRC5A is involved in the regulation of STAT3 signaling pathway. Knockdown of GPRC5A correlates with STAT3 activation in cancers such as lung cancer and head and neck squamous cell carcinoma (HNSCC), pointing to a tumor suppressive role for GPRC5A. Compared to GPRC5A wild-type cells, GPRC5A knockout cells have higher levels of activated-STAT3 and STAT3-regulated anti-apoptotic genes, independent of the presence of exogenous epidermal growth factor (EGF), resulting in enhancement of tumor progression [66, 67]. Contrarily, another study indicates that GPRC5A is positively correlated with STAT3, and that* GPRC5A* silencing is associated with suppression of STAT3 phosphorylation at TYR705 in human pancreatic cell lines [68]. These data suggest that in some cases GPRC5A may play an oncogenic role by activating STAT3 signaling and in others has a tumor suppressor role through STAT3 phosphorylation inhibition.

### 4.4. Focal Adhesion Kinase (FAK)/Src Signal Pathway

The regulation of cell-cell and cell-matrix adhesion plays a vital role in the integrity and homeostasis of epithelial tissue [69, 70], and interference with this process may contribute to tumor progression. The most important function of the FAK signal pathway is regulating cell adhesion [71–74]. GPRC5A silencing deregulates integrin *β* 1 (ITGB1) expression leading to restrained capacity of integrin-mediated cell adhesion. GPRC5A knockout interferes with the activation of the FAK/Src signaling pathway and the activity of downstream RhoA and Rac1 small GTPases [75].

## 5. GPRC5A and Its Role in Human Cancer

Although GPRC5A is predominately expressed in normal lung tissues, dysregulation of GPRC5A expression has been observed in a variety of human cancers (Table 1).

### 5.1. GPRC5A and Lung Cancer

GPRC5A exhibits a promising tumor suppressive role in lung cancer. Its expression, both at the mRNA and protein level, is much lower in lung cancer than in healthy lung tissue [66, 76–78]. According to recent reports, the expression of GPRC5A is the highest in disease-free normal bronchial epithelia (NBE), intermediate in cancer-free lungs from patients with chronic obstructive pulmonary disease (COPD) and the lowest in patients with COPD and lung cancer [77]. Moreover, homozygous GPRC5A knockout mice are more likely to spontaneously develop lung tumors than GPRC5A heterozygous or wild-type mice, with tumor incidence rates of 76%, 11%, and 10%, respectively [78].* In vitro* experiments demonstrated that overexpression of GPRC5A inhibits cell viability and colony-formation and enhances apoptosis in NSCLC cell lines [31, 56, 66, 78]. Similar results were found in another study, which reported that lung epithelial cells from* GPRC5A* wild-type mice have worse viability and colony-formation ability than lung cells from GPRC5A knockout mice [56]. Importantly, the effect of GPRC5A knockout on lung tumorigenesis can be strengthened by tobacco-specific carcinogen nicotine-derived nitrosamine ketone (NNK). The NNK-treated group developed lung adenocarcinoma sooner than the saline-treated control group, an effect which was most likely enhanced by mutations in multiple genes, such as those for ATM, histone methyltransferase 2D (KMT2D), neurofibromatosis type 1 (NF1), transformation related protein 53 (Trp53), MET, and enhancer of zeste homolog 2 (Ezh2) [79, 80]. Several parallel studies showed that GPRC5A exerts its tumor suppressive effect by regulating the NF-*κ*B and EGFR/STAT3 signaling pathways. Compared to wild-type cells, the NF-*κ*B signaling pathway is activated in GPRC5A knockout cells, which contributes to lung inflammation and tumorigenesis. These effects can be reversed by silencing of the P65 subunit of NF-*κ*B [79, 81]. Additionally,* GPRC5A* knockout enhances the transformed phenotype in normal and tumor cells through the aberrant activation of the EGFR/STAT3 signaling pathway [66]. Interestingly, there is a mutual effect between EGFR and GPRC5A. On the one hand, EGF induces TYR phosphorylation on the C terminal of GPRC5A, resulting in the suppression of GPRC5A-mediated inhibition of cell invasion and anchorage-independent growth of NSCLCs [45]. On the other hand, GPRC5A interacts with EGFR through its 7TM domains, leading to the activation of EGFR/STAT3 signaling pathway and its downstream target genes, preventing spontaneous and ionizing radiation-induced lung tumorigenesis [82].

### 5.2. GPRC5A and Breast Cancer

Elevated GPRC5A mRNA expression has been observed in breast cancer cell lines and clinical tumor tissues (25 primary breast cancer tissues), and GPRC5A knockdown leads to inhibition of cell growth in cell lines MCF7 and T47D [83]. Similar results have been obtained in 293 cells (HEK-293 F cells) which exhibited augmented anchorage-independent growth ability upon GPRC5A ectopic expression [30]. Furthermore, GPRC5A together with FXYD domain-containing ion transport regulator 3 (*FXYD3*) and* PYCARD* have been reported as potential predictors of pathological grading of breast cancer and might benefit the management of clinical treatments [84]. However, immunohistochemical (IHC) analysis of a tissue microarray consisting of 147 invasive breast cancer samples and 44 normal breast tissue samples showed that GPRC5A is abundantly expressed in breast cancers, whereas no association was discovered between GPRC5A expression and clinicopathological characteristics [85]. Additionally, knockout of* GPRC5A* results in reduced cell adhesion and spreading ability, via deregulation of ITGB1 expression and suppression of FAK/Src signaling [75]. All these results reveal a tumor-promoting role of GPRC5A in breast cancer. However, one early study suggested that GPRC5A exhibits a tumor-suppressive role in EGFR-expressing MDA-MB-231 cells and that GPRC5A knockdown promotes colony formation, cell growth, cell migration and invasion capacities in this cell line, but has no such effect in EGFR-negative MCF7 cells. Specifically, GPRC5A knockdown augmented EGF signaling, an effect which can be reversed by inhibiting EGFR phosphorylation [86].

### 5.3. GPRC5A and Colorectal Cancer

GPRC5A is highly expressed in colorectal cancer (CRC), and elevated GPRC5A expression is significantly associated with inferior prognosis [87, 88]. In addition, liquid chromatography analysis demonstrates that GPRC5A expression is lower in polyps than in metastatic and non-metastatic CRC samples, suggesting that GPRC5A may serve as a biomarker to differentiate CRC from normal tissues [89]. What is more, GPRC5A deficiency reduces cell proliferation and promotes cell apoptosis* in vitro* and inhibits tumorigenesis of a colitis-associated cancer model* in vivo*. Furthermore, GPRC5A can be induced by hypoxia, regulates the NF-*κ*B-mediated expression of Vanin-1 (a key enzyme of cysteamine generation), and influences the reactive oxygen levels contributing to tumor progression [88, 90].

### 5.4. GPRC5A and GC

GPRC5A is expressed in the membrane of cells in gastric tissues. Compared to normal gastric tissues, GPRC5A mRNA and protein expression levels are significantly elevated in GC tissues [91]. Increased GPRC5A expression is significantly related to aggressive clinical parameters (larger tumor size, diffuse type, serosal invasion, and lymph node metastasis) and shorter overall survival (OS) [92].

### 5.5. GPRC5A and Hepatocellular Carcinoma

Conflicting information exists concerning GPRC5A's expression status in hepatocellular carcinoma (HCC). Lower GPRC5A mRNA levels have been reported in seven cell lines established from patients-derived tumor xenografts [93]. Conversely, several studies found that GPRC5A expression is elevated in HCC compared to in paratumor and normal liver tissues, and high GPRC5A expression is related to advanced clinical stage, high serum alpha-fetoprotein (AFP), vascular invasion, tumor recurrence, and worse prognosis (OS and disease-free survival) [94, 95].

### 5.6. GPRC5A and Pancreatic Carcinoma

GPRC5A expression is generally low in normal pancreatic ductal cells but is dramatically increased in pancreatic ductal cells of primary and metastatic tumor samples [37, 96]. Knockdown of* GPRC5A* with siRNAs leads to morphological changes in pancreatic tumor cells AsPc-1 [30]. Suppression of GPRC5A impaired the cell growth, proliferation, colony formation, and migration ability of pancreatic ductal adenocarcinoma cells [37, 68, 96].

### 5.7. GPRC5A in Other Cancers

In intrahepatic cholangiocarcinoma, GPRC5A is up-regulated compared to normal controls [95]. In oral squamous cell carcinoma (OSCC), GPRC5A is downregulated compared to normal oral epithelium, and this downregulation is associated with poorly differentiated OSCCs. Consistently, GPRC5A overexpression reversed the malignant phenotype of OSCC cell lines, implying that GPRC5A may serve as a powerful biomarker for malignant OSCCs [97]. HNSCC is associated with suppressed expression of GPRC5A, which is positively associated with tumor grade, along with the activation of STAT3. Overexpression of GPRC5A suppressed interleukin (IL)-6-induced STAT3 signaling pathway activation and inhibited colony-formation in HNSCC cells [67].

## 6. Clinical Application Value of GPRC5A

As described above, GPRC5A is dysregulated in a broad range of cancers, which indicates that it can potentially be used as a diagnostic candidate, especially in lung cancer. Further large-scale studies are therefore warranted to evaluate its diagnostic sensitivity and specificity in different cancer types. Moreover, GPRC5A, as a member of the largest family of protein targets for approved drugs (GPCRs) [98], is also a potential therapeutic target in patients with elevated GPRC5A levels. Until now, only tretinoin (ATRA, DB00755) has been demonstrated to be related to GPRC5A, whereas its role in antitumor therapy remains unknown. Future studies are therefore urgently warranted. Notably, GPRC5A also has great values in the optimization of clinical medication. In pancreatic cancer, suppression of GPRC5A was found to increase the cell sensitivity to multiple chemotherapeutic drugs, including gemcitabine, oxaliplatin, and fluorouracil [37, 96]. Additionally, EGFR inhibitors have been shown to be more effective in GPRC5A knockout lung cancer cells than in GPRC5A wild-type lung cancer cells, indicating that they are more suitable for lung cancer patients with lower GPRC5A expression [76]. Therefore, despite few studies having focused on its clinical application, GPRC5A's importance is clear as it could benefit accurate diagnosis and it should be taken in consideration for targeted-therapies and optimizing clinical medications.

## 7. Conclusions

**Figure 2:**
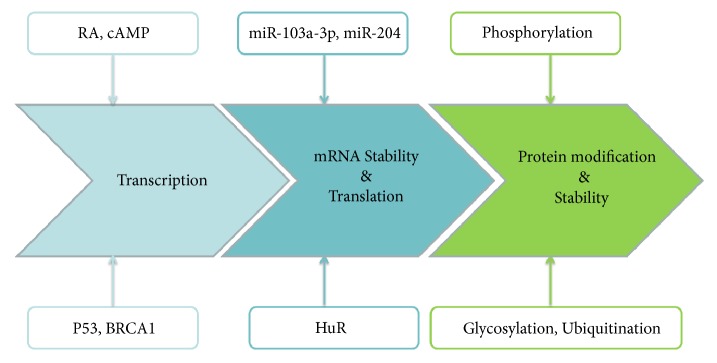
The regulation of GPRC5A expression in cancer cells. GPRC5A is subjected to multiple levels of regulation from transcription to translation as detailed in the text.

The lack of effective biomarkers for early diagnosis and lack of valid therapeutic methods for the treatment of aggressive cancers are the most intractable issues in clinical cancer management. GPRC5A is a member of orphan class C of the GPCR superfamily and was originally identified as a tumor suppressor playing an important role in lung tumor development. The* GPRC5A* gene contains many binding sites for transcription factors: this allows the regulation of GPRC5A expression by RA, cAMP, BRCA1, and many others. Additionally, GPRC5A expression is regulated posttranscriptionally and posttranslationally (Figure 2). Accumulating studies have demonstrated GPRC5A dysregulation in various human cancers, although its expression status differs among different cancer types. Aberrant GPRC5A expression induces the deregulation of signaling pathways such as cAMP, NF-*κ*B, STAT3, and FAK/Src signaling and is related to prognosis. Especially, GPRC5A expression is associated with a poor response rate to chemotherapy. These data suggest that GPRC5A can be regarded as a potent biomarker for accurate diagnosis, prognosis prediction, and personalized treatment for patients with cancer. However, current knowledge of the exact mechanism of these processes is limited. Further studies focused on the cellular and molecular mechanisms will reveal novel insights into the details of its intricate function in cancer.

## Figures and Tables

**Table 1:** The dysregulation of GPRC5A in human cancers.

Study	Year	Tumor type	Kind/num. of analyzed samples	Methods	Expression status	Potential clinical significant
Nagahata, T.	2005	BC	25 primary BC tissues	qRT-PCR	up	None

Tao, Q.	2007	LC	18 pairs of human LC tissues and adjacent normal tissues; microarray with 186 LC tissues and 17 normal lung samples	qRT-PCR; Microarray	down	None

Dairkee, S. H.	2009	BC	50 pairs of cDNAs from matched BC and normal breast tissues; 147 invasive BC and 44 normal breast tissues	CPA;IHC	up	No association with clinical parameters and prognosis

Cheng, L.	2012	GC	25 paired GC tissues and matched adjacent non-tumor tissues	Microarray; qRT-PCR	up	None

Fujimoto, J.	2012	NSCLC	31 NBE,24 COPD,26 COPD with cancer and 474 NSCLC tissnes;6 NSCLC and matched normal lung tissues	Microarray; qRT-PCR	down	Positively associated with adenocarcinoma histology; highly suppressed in NCSSLC

Liu, S. L.	2013	OSCC	60 paired primary OSCC and adjacent normal specimens	IHC	down	Inversely correlated with the malignant grade

Subrungruanga, I.	2013	ICC	18 paired ICC and matched normal tissues	Microarray; qRT-PCR	up	None

Zougman, A.	2013	CC	347 CC specimens	Microarray	up	Positively correlated with tumor recurrence

Kume, H.	2014	CRC	33 primary CRC and 16 colon polyps	LC-MS/MS	up	None

Lin, X.	2014	NSCLC	129 paired NSCLC and adjacent normal tissues	IHC	down	None

Sokolenko, A. P.	2014	BC	17 BC with BRCA1 mutation and 94 BRCA1 non-mutation tissues	qRT-PCR	down in BRCA1 mutation samples	Inversely correlated with BRCA1 mutation

Zheng, J.	2014	HCC	106 HCC	qRT-PCR;WB; IHC	up	Positively correlated with advanced TNM stage, high serum AFP, vascular invasion, tumor recurrence, DFS and OS

Liu, H.	2016	GC	30 paired GC and adjacent normal tissues;106 GC samples	qRT-PCR;WB; IHC	up	Positively associated with tumor size, diffuse type, serosal invasion,lymph node metastasis and OS

Zhou, H.	2016	Pancreatic cancer	46 normal pancreatic tissues,145 primary pancreatic tumors and 61 metastatic tumors;203 samples pancreatic tumors	Microarray; IHC	up	None

Jahny, E.	2017	PDAC	435 PDAC and 209 non-cancerous pancreatic tissues	Microarray	up	None

Liu, S.	2017	HNSCC	86 paired HNSCC and adjacent normal tissues	IHC	down	Positively associated with tumor differentiation

Zhang, L.	2017	CRC	57 paired CRC and adjacent normal tissues	qRT-PCR	up	Positively associated with tumor grade

BC: Breast cancer; LC: lung cancer; GC: gastric cancer; NBE: normal bronchial epithelia; COPD: chronic obstructive pulmonary disease; NSCLC: nonsmall cell lung cancer; OSCC: oral squamous cell carcinoma; ICC: intrahepatic cholangiocarcinoma; CC: Colon cancer; CRC: colorectal cancer; HCC: hepatocellular carcinoma; PDAC: pancreatic ductal adenocarcinoma; HNSCC: head and neck squamous cell carcinoma; qRT-PCR: quantitative real-time PCR; CPA: cancer profiling array; IHC: immunohistochemistry; LC-MS/MS: liquid chromatography with tandem mass spectrometry; WB: Western blot; DFS: disease-free survival; OS: overall survival.
